# Development of an Innovative Mechatronic Binder Machine

**DOI:** 10.3390/s22030741

**Published:** 2022-01-19

**Authors:** João Sousa, Luis Figueiredo, Carlos Ventura, João Pedro Mendonça, José Machado

**Affiliations:** 1 MEtRICs Research Center, Campus of Azurém, University of Minho, 4800-058 Guimarães, Portugal; jsousa@dem.uminho.pt (J.S.); luisfilipe_89@hotmail.com (L.F.); jpmas@dem.uminho.pt (J.P.M.); 2 Acco Brands Portuguesa Lda, Zona Industrial Paço, 4970-249 Arcos de Valdevez, Portugal; carlos.ventura@acco.com

**Keywords:** mechatronics, binding, sensorization, servitization

## Abstract

This paper describes the development of a mechatronic punch and bind office machine. Integrating smart technologies in the existing traditional business machines will ease the evolution of these systems, enabling productivity and efficiency. The development of an experimental platform that enables further advances in servitization is required. To increase the binding rate of the office document, as well as to reduce the likelihood of errors, efforts have been made to develop a measuring system that allows the document to be properly measured and specifies the appropriate binding spine at the same time. As a complement, developments have been conducted in a system that enables the verification of the inserted spine. In addition, a system for automated document binding along with an integrated platform that allows the communication between all systems is presented. In both its hardware design and its underlying sensors, the new system has several advantages, providing significant performance improvements and upgradability over existing systems. This alternative comprises a system that enables a variety of sheets of paper, plastic or other materials to be punched.

## 1. Introduction

### 1.1. Related Work

In recent years, offices have introduced many technological developments, which have had a huge effect on the work setting and productivity. These techniques include physical machines such as laptops, multimedia systems, intelligent boards, digital programs, intranets, etc. Traditional equipment such as binders, staplers, etc. need to develop with the growth and application of these technologies, as well as increasing productivity needs in offices.

This design seeks to automate the machinery, increase the production rate, eliminate errors and facilitate the use of the equipment. Several kinds of plastic or metal spines can be used to bind paper documents, plastic or other materials. Binding equipment used to bind documents relies on the selected spine as the binding process depends on the form and material of the spine. There are currently a broad variety of punching and binding options available for both plastic and metal spines, which may have a manual or automatic actuation. Research and development attempts have been made in recent years to boost the punching ability of the equipment [1,2,3,4]. These attempts stem from the need to divide a document into smaller subdocuments, the thickness of which can be punched through. When it comes to document binding developments, they have not been so capacity-driven, highlighting Scott Cox’s innovations [5] as well as the innovations of Gary Badham and Gareth Owen Morgan [6]. These innovations have been focused on mechanical machinery, enabling some mechanism automation. However, it remains the user’s responsibility to control the different binding and insertion parameters of punched documents or subdocuments in the spines.

Of the various existing spines, we highlight the ProClick spine [7] type, which provides a permanent and secure binding in addition to allowing successive reuse and subsequent recycling. The focus of this paper, i.e., describing an innovative strategy to a mechatronics prototype, is based on the use of such spines on a punching and binding device. Some innovations to binding machines have been made using this type of spine, such as those developed by Tomaoki Sakata and Toru Yoshie [8], Colin Knight [9], and Robert Jervis, Michael James and Robert Hadden [10].

These innovations referred to are solely mechanical, as they have binding malfunctions attributed to improper use, the enhanced punching and binding productivity is small, and there are no developments regarding the servitization of office equipment.

Some devices such as the P2000 and P3000 [11] already have automation in both punch and bind systems, as well as the ability to assist users with certain binding process-related choices. Although these systems already have automation, they still make many mistakes during punching and binding and are still below the developments created in other office equipment. Examples of punching and binding mistakes include the following: (a) selecting the incorrect comb, spine, or coil due to the incorrect measurement of the document’s thickness; (b) punching with a misaligned document; or (c) failing to insert all the elements of the comb, spine, or coil in the punched holes. Therefore, there is a need to develop automatic devices to guarantee correct punching and binding of a document, boost productivity, decrease the user interference, and check the potential for user errors. To fulfil the goals of Industry 4.0, such as improved communication, reliability and safety, the developed inventions serve as a basis for a subsequent evolution [12].

In this manner, this study is intended to develop an automated prototype capable of binding documents; point out the optimal spine for use in the binding method; check whether the inserted spine for binding is suitable for binding; adjust the document height autonomously; check the sheet alignment for punching; automatically punch and bind, ensure that user-induced binding mistakes are eliminated; ensure ideal binding; and increase punching and binding productivity.

### 1.2. Equipment Use Description

Punching and binding equipment can be operated manually or by using a motor. Only the punching mechanism is automated in most automatic machines. This is because a big quantity of force is needed to punch a big number of sheets. Moreover, compared to the binding mechanism, the punching mechanism is much easier. The user starts by selecting the spine to be used to bind; this action is generally supported by a sticker positioned on the device. The user then counts the document sheets, separating them into subdocuments with a maximum number of sheets corresponding to the punching capability of the device (generally 15 sheets). The subdocuments can be placed in the punching slot after this action and the punching mechanism can be activated manually or automatically. The customer checks if the punch is well conducted at the end of each punched subdocument. This action is the responsibility of the user and great care is required to prevent errors by placing the sheets in the punching slot. The sheets are combined into a single document when all subdocuments are punched, and then a spine is used to bind the document. There is a mechanical mechanism to assist the binding process in most presently accessible machinery. This mechanism usually keeps the spine open or helps to close it. The sheets must be inserted by the user in the spine, a process that is time-consuming and prone to mistakes. The mechanism was documented by Jervis, James and Hadden [10]. This system enters the fingers of the spine into the document’s holes and closes the spine [9]. It facilitates the binding process, as it is the duty of the user to adjust two side tabs to shift the document’s binding height. If the spine is wrongly selected, or if the tabs are not adjusted properly, the binding fails. The paper is bound when all these activities are completed. It is recognized that when using the prototype, the user can punch and bind the paper sheets in a single document. The prototype was created to efficiently enable these activities. An algorithm was developed to control the prototype and reduce the interaction of the user in the punch and binding process for the proper implementation of these tasks.

This paper is organized as follow: Section 1 serves as an introduction to the subject; Section 2 describes the prototype requirements and main systems; Section 3 describes the control and electronic systems of the prototype; Section 4 describes the control algorithm of the prototype and their mechanisms, which are presented in Section 2; and Section 5 presents a conclusion of this work.

## 2. BindTronic Prototype

### 2.1. Prototype Requirements

This section is intended to present the prototype requirements and the systems created to fulfil the requirements needed. Considering the different tasks required for the prototype (punching and binding), three mechatronic systems have been developed. A punching mechatronic device has been created to pierce up to 25 sheets of paper in one block for paper punching. A mechatronic system was developed for sheet binding, and is capable of automatically adapting to the size of the spine and the number of sheets used, performing a perfect binding. This system needs to be able to close the three existing ProClick spine sizes. To allow the binder system to be automated, the thickness of the binding document (number of sheets) must be known. Therefore, a mechatronic device has been created to measure this data. The integration of these three mechatronic systems, including structural support and system control, includes the practically autonomous prototype, requiring the user to only bring the paper sheets into the three advanced devices and choose the activities he/she wishes to carry out on a touchscreen. The activities of the user are always verified to guarantee that the paper is perfectly printed and bound.

**Figure 1:**
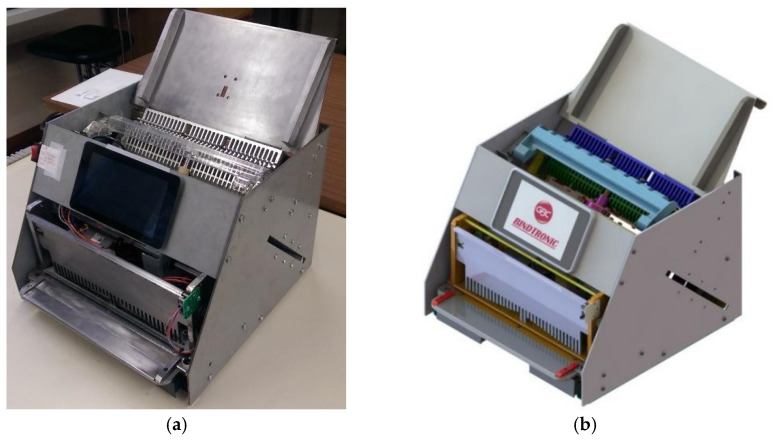
BindTronic prototype: (**a**) prototype; (**b**) 3D CAD model.

The BindTronic prototype (Figure 1) was developed to be an autonomous prototype, capable of measuring the thickness of the document to be bound and indicate the ideal spine. It can check if the inserted spine allows the measured document to be bound, preventing binding errors; adjust the document height in an autonomous and automatic way, according to the spine used; verify the alignment of the sheets to be punched, as well as the capacity of the waste drawer; eliminate user errors by performing the punch and bind automatically; and increase the productivity of both punching and binding.

To fulfil the described objectives, three separate mechatronic systems were developed. For paper punching, a mechatronic system capable of punching 34 square pins with a capacity of up to 25 sheets was developed. For the binding, a mechatronic system was developed that automatically adapts to the document and spine size, guaranteeing a perfect binding. This system can bind the three spine sizes currently available on the market and can automate the document binding process. Moreover, a measuring system to measure the document’s thickness was developed.

The BindTronic prototype is constituted by the integration of these three developed mechatronics systems, along with the structural support and control system. In this prototype, the user only needs to put the sheets from the block of paper and spines in the various systems and choose the actions that they want to perform on the touchscreen.

**Figure 2:**
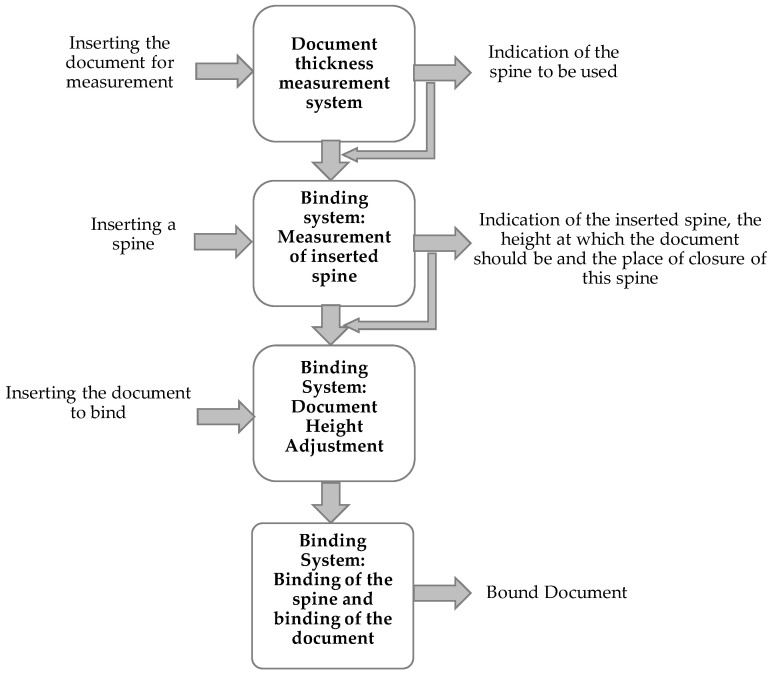
Diagram of the interaction of the different systems and mechanisms that interact to make a binding.

Through the flowchart in Figure 2, it is possible to verify the complete functioning of the developed prototype, as well as the various windows with information for the user that appear on the touchscreen. To facilitate the understanding of the general functioning of the prototype, we present an interaction scheme of the different systems that constitute the prototype, as well as two block diagrams that illustrate the prototype’s operation. In Figure 2, a diagram showing the interaction of the user and the various systems and mechanisms for binding is shown.

The interaction diagram is only presented for the binding system, since the punching system is an isolated system; there is no interaction with other systems or mechanisms of the prototype for its operation.

In Figure 2, the arrows on the left represent actions that are performed by the user and the arrows on the right represent the information provided to the user. Some of this information is also used by the control system to know the actions it needs to perform. The spine thickness measurement system requires the user to enter a document, subsequently providing document size information corresponding to the existing spine sizes. In the binding system, the sprue metering mechanism requires the user to insert a binding spine, verifying whether the inserted spine allows for the binding of the document, by comparison with the information of the previous one. In the mechanism of the measurement of the inserted spine, there is also information provided on the height the document must be to be bound with the chosen spine, as well as the place of closing of the spine. In the document height adjustment mechanism, the binding document is inserted, and the information from the previous mechanism is used to adjust the height of the document. Finally, the mechanism that closes the spine is used, and this mechanism also uses the information from the first binding system to know the distance that needs to be travelled to close the spine and bind the document.

**Figure 3:**
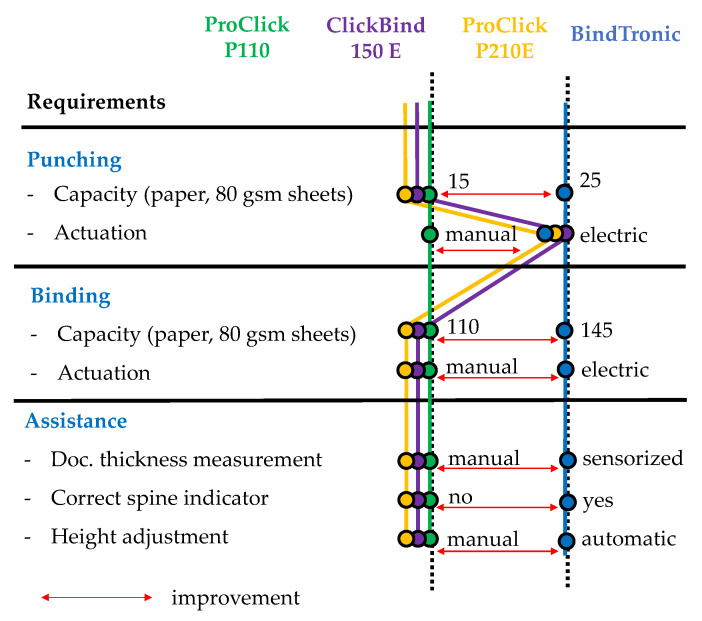
Different ProClick binding profiles and BindTronic improvements.

Figure 3 shows how the design for the BindTronic prototype compares to previous ProClick binding machines. The prototype corresponds to a completely different machine profile with respect to punching, binding and user assistance. When compared to previous machines, BindTronic improves the punching capacity from 15 to 25 paper sheets (80 gsm). The plastic cover punching of more than two units at a time was not tested since it is not a requirement for improvement. Binding capacity was also improved, due to the document height adjustment mechanics that avoid misalignments when closing the spine. Other user assistance features, such as sensorized document thickness measurement, the already mentioned automated height adjustment and a correct spine indicator, significantly shorten the punch and bind cycle and reduce operator errors.

### 2.2. Document Thickness Measurement System

**Figure 4:**
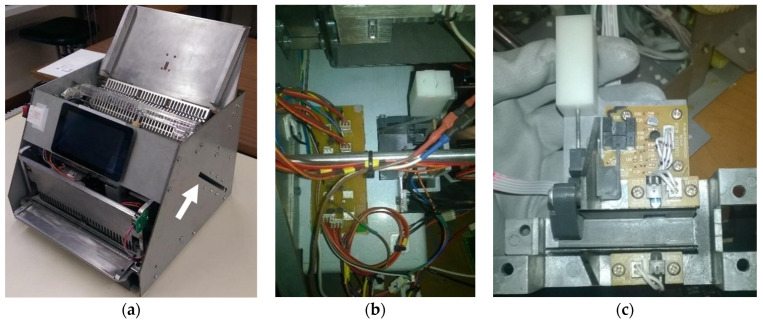
Mechatronic system to measure the document thickness: (**a**) lateral groove in the structure; (**b**) metal structure inside the groove with electronic components attached; (**c**) document thickness measurement mechanical and electronic components.

The document thickness measurement system is shown in Figure 4. This system allows for measuring the document in three different sizes.

**Figure 5:**
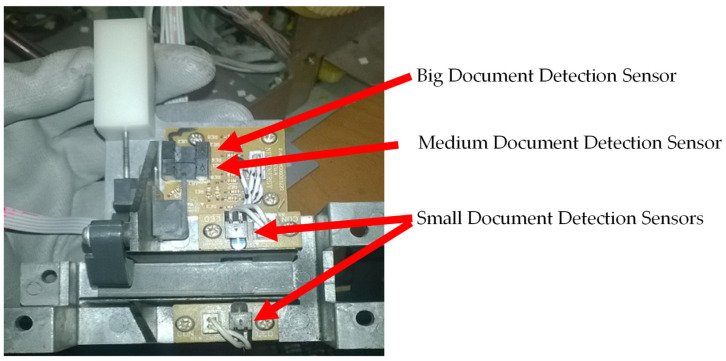
Document thickness measurement system showing which sensor indicates which size.

A groove in the lateral structure of the prototype (Figure 4a) has been developed to insert the document for measurement. This document is guided by another metal structure (Figure 4b) to the measurement system (Figure 4c). The measuring system uses three optical sensors and a cam system with a return spring. The information given by this system indicates the document size in relation to the three existing spine sizes. The document inserted for measurement will actuate the cam, which undergoes a displacement proportional to the thickness of the document. If only one sensor is activated, i.e., the light path between the LED and the phototransistor of the small document detection sensors (Figure 5) blocked by the document, the document is of small size; if two sensors are activated (i.e., the small document detection sensors and the medium document detection sensor are blocked), the document is of medium size. In the case of a large document, all sensors are activated. Thus, for all three scenarios, the small document detection sensors are always blocked by the measured document.

In Figure 5, it is possible to observe the document thickness measurement system with the sensor’s reference. The description of the connections with the Raspberry Pi are indicated, showing to which GPIO pins the sensors are connected.

### 2.3. Binding System

The binding system is a complex system, and is composed of three different mechanisms that work together to perform the binding. These mechanisms measure the inserted spine for binding, the mechanism that adjusts the height of the document to be bound, and the mechanism that closes the spine. The displacements provided by the document height adjustment mechanism and the displacements performed by the spine closing mechanism, depend on the inserted spine. In the case of the document height adjustment mechanism, the larger the inserted spine for binding, the higher/farther the spine must be from the document so that the spine fingers enter the perforations of the document. In the spine closing mechanism, the larger the spine, the less this mechanism has to perform displacement to close it.

#### 2.3.1. The Inserted Spine Measurement Mechanism

**Figure 6:**
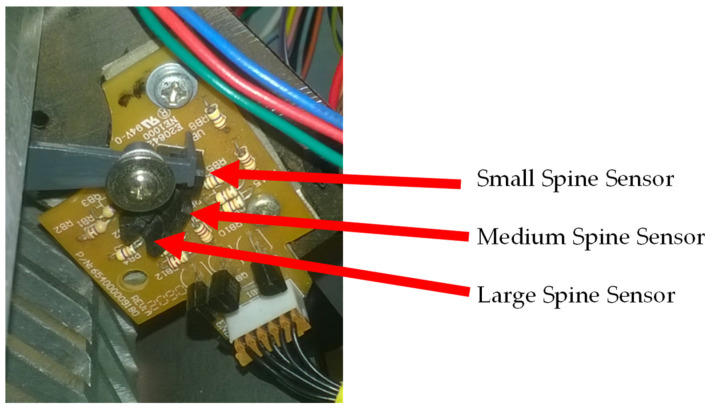
Inserted spine measurement mechanism indicating the size of each spine.

In Figure 6, it is possible to observe the electronic board and sensors that make the measurement of the inserted spine. This system has a tab that works similar to a lever. The tab is placed where the spines are inserted. As they are inserted into the binding system, the spines push the tab at one end, causing its other end (the grey part in Figure 6) to shift, activating one of the three optical sensors. The larger the size of spine inserted, the greater the shift of the tab, causing it to activate a sensor farther from the tab’s resting place. This system does not prevent the binding of the spine since the tab is pushed by the part that closes the spine.

#### 2.3.2. Document Height Adjustment Mechanism

The control system performs the spine reading measurement through the inserted spine measurement mechanism. This information is used to automatically adjust the height of the document relative to the spine closing mechanism. As stated, the larger the spine inserted for binding, the greater the distance the document needs to be in relation to the spine closing mechanism. This is so that it is possible for the spine fingers to enter the perforations of the document, thus resulting in the need to have a system that adjusts the height of the document.

**Figure 7:**
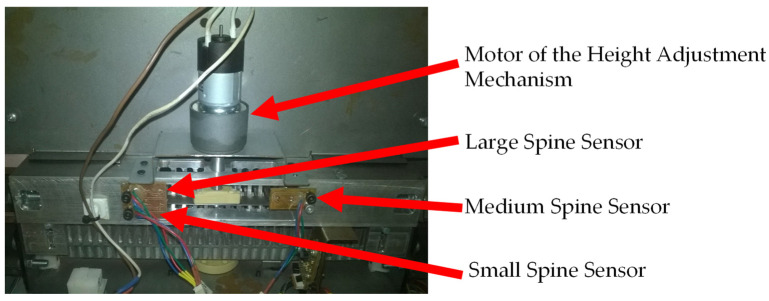
Document height adjustment mechanism showing which sensor indicates the height for different spine sizes.

This system has three sensors, each one representing the ideal height to bind the three ProClick spine sizes. In Figure 7, it is possible to observe the arrangement of the PCB boards with the sensors, as well as the indication of which sensor corresponds to the height of each spine (small, medium and large).

**Figure 8:**
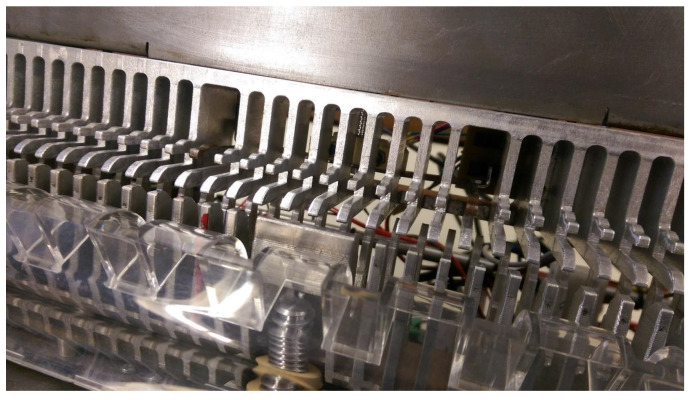
Pins used in the document height adjustment mechanism placed at the height for binding with a large spine.

The operation of this system is performed by means of an electric motor visible in Figure 7. The motor is coupled to a screw that raises and lowers the parts that hold the document. The document to be bound is placed in a part with several pins to hold it. The pins are placed so that the prototype is ready to bind documents with small spines. For document bindings with medium or large spines, it is necessary to raise the pins, and in this prototype, only two pins are coupled to the nut and to the screw, allowing the elevation of the document (Figure 8).

#### 2.3.3. Spine Closing Mechanism

**Figure 9:**
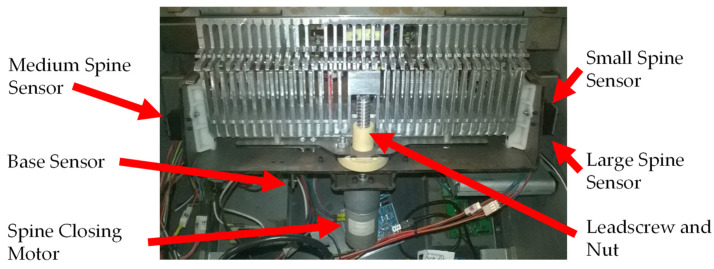
The spine closing mechanism indicating the sensors used for closing spines of each size.

The spine closing mechanism (Figure 9) is the last mechanism of the binding system. This mechanism is responsible for closing the three sizes of ProClick spines. The integration of these three mechanisms makes it possible to perform automatic binding, where the user only has to place the spine, insert the document to be bound and give the command to start the binding process.

In addition to the three sensors that indicate the location of the closure of each spine size, this mechanism also has a sensor that indicates the base or initial position of this system (Figure 9). The sensor that marks the base or initial position is necessary because this mechanism needs to be fully retracted to permit the insertion of a spine and to permit the removal of a document already bound. For the actuation of this mechanism, an electric motor is used, which is coupled to a leadscrew and nut responsible for the closing of the spine. This mechanism action is similar to the document height adjustment mechanism.

### 2.4. Punching System

**Figure 10:**
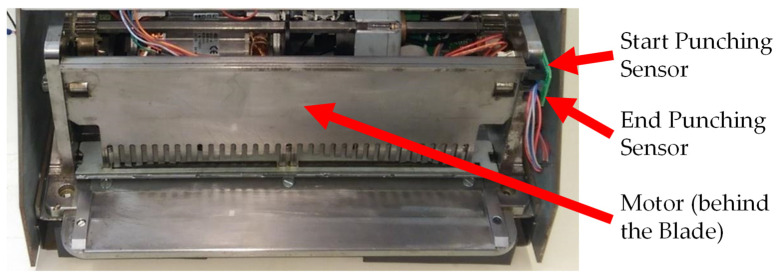
Punching system.

The punching system for the BindTronic prototype is similar to others from ACCO Brands Corporation and is shown in Figure 10.

The punching system of the prototype uses a blade with 34 square punches of 4 mm by 4.5 mm and is actuated by an electric motor. This system has two sensors to indicate the start and end position of the blade.

In addition to these sensors, the waste bin sensor (not visible in the image) can also be associated with this system. This sensor is used to check if the waste bin is inserted, and to reset the punch counter when cleaning the waste bin drawer. The user can choose whether to use a waste bin, and the control system only allows binding if the option chosen matches whether the waste bin drawer is placed. The reset to the punch counter that checks the capacity of the waste bin has been developed to reset if the waste bin drawer is removed for more than 5 s (minimum time allowed for cleaning).

### 2.5. Control System

The software for the BindTronic project runs in the control system of the prototype. This control system aims to read the various sensors available on the prototype and respond accordingly to the actions intended by the user. It also has the function of checking if the choices and selections that the user is using are correct, in order to prevent errors during document punching or binding.

**Figure 11:**
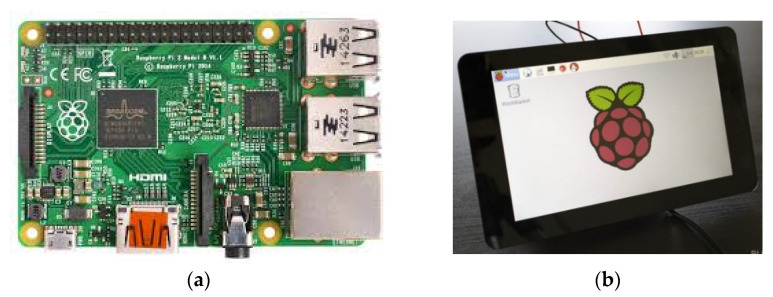
Control system: (**a**) Raspberry Pi 2B; (**b**) touchscreen.

For the control system of the prototype (Figure 11), a Raspberry Pi 2B and a touchscreen were used for control and interaction with the users.

The BindTronic prototype does not have buttons for interaction with the user, and it is from the touchscreen that the user receives information from the control system and interacts with it, selecting the options and actions he/she wants to perform.

To be able to control the various mechanisms of the prototype, GPIO (general purpose input/output) pins are used, and they connect the sensor circuits and motor circuits to the control system. The information is collected from the sensor circuits, and actions are carried out in the motor drive circuits according to the user’s selections.

## 3. Electric Connections Scheme

In this section the schemes, electrical connections and some characteristics corresponding to the electronic hardware used in the prototype of the BindTronic project are presented.

### 3.1. Raspberry PI GPIO Connections

**Figure 12:**
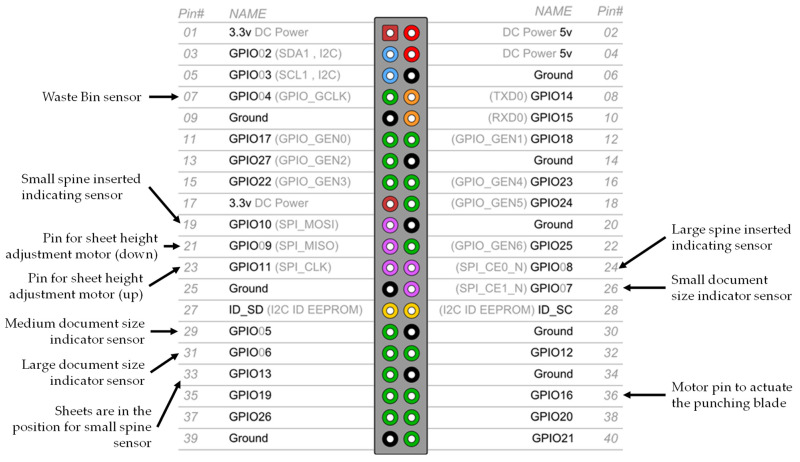
Connection definitions of Raspberry Pi GPIO. Note: Optical sensors and their respective circuits indicate that an object is present when there is NO light transmission between the transmitter and the receiver, by setting its GPIO pin to the low logic level (0 Volts). If there is a problem or a lack of connection with the sensors or their circuits, the GPIO will have the pull-up level. In the case of pull-up to low, it will indicate to the program that the moving parts are interrupting the sensor. In the case of pull-up to high, it will indicate to the program that there is light transmission (even if it does not exist, or in the case of a problem). These GPIOs must be used in situations that do not cause any problems.

In Figure 12, the GPIO pins available in Raspberry Pi 2B are represented, as well as their alternative functions (not used in the software developed). It also shows which GPU pins of Raspberry Pi 2B were used, and for which function they are used in the prototype and software, thus representing which circuits or sensors they are connected to.

### 3.2. Definitions and Electrical Schematics for the Sensors

In this section, we present the types of sensors used, their electrical diagrams, drawings of the PCB boards and images with a demonstration of the corresponding connections realized in the prototype. Two types of sensors were used in the BindTronic prototype, optical sensors and mechanical sensors.

#### 3.2.1. Optical Sensors

Most of the sensors used in the BindTronic prototype were optical sensors due to their reliability, reduced cost and large number of life cycles.

Table 1 shows the optical sensor used, as well as indicating the annex corresponding to its datasheet with detailed information about it.

**Figure 13:**
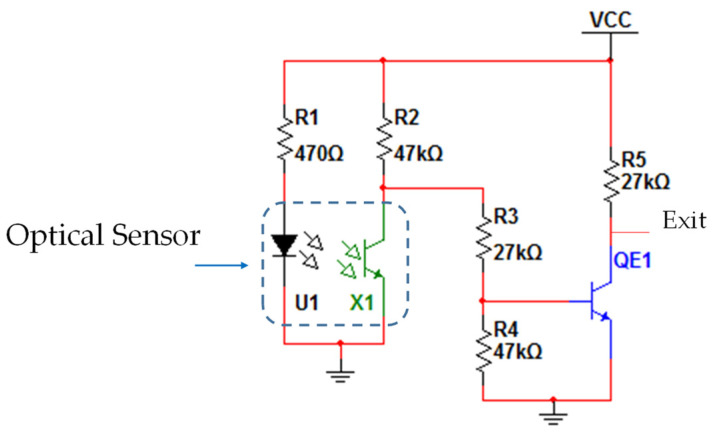
Electric schematic for the optical sensor.

Figure 13 shows the electrical scheme used with the optical sensors.

**Figure 14:**
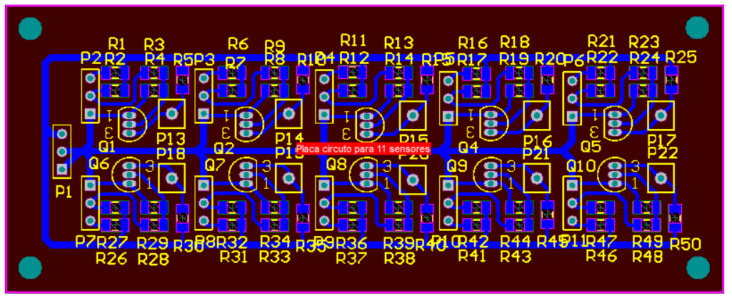
Board with ten electrical circuits for connections to optical sensors (drawing in the bottom layer).

The electrical circuit where the sensors are connected (Figure 13) serves to power the optical sensors as well as to convert their analog signal to a digital signal (0 or 1). If any object interrupts the transmission of light between the transmitter (LED) and the receiver (phototransistor) of the optical sensor, there are zero volts in the circuit output (0 volts) corresponding to the logic level 0. The logic level 1 (VCC) is present at the circuit output when there is light transmission between the LED and the phototransistor of the optical sensor. Figure 14 shows the design of a PCB board developed with ten electrical circuits equal to the one in Figure 13; it was developed to connect ten optical sensors.

Power supply to the electrical circuits is carried out via the P1 connector, where pins 1 and 2 are used for the ground connection, and pin 3 for the connection to the VCC (3.3 V). The sensors are connected to the remaining three-pin plugs (P2 to P11) and the result of each sensor is removed from the one-pin plugs (P13 to P22).

**Figure 15:**
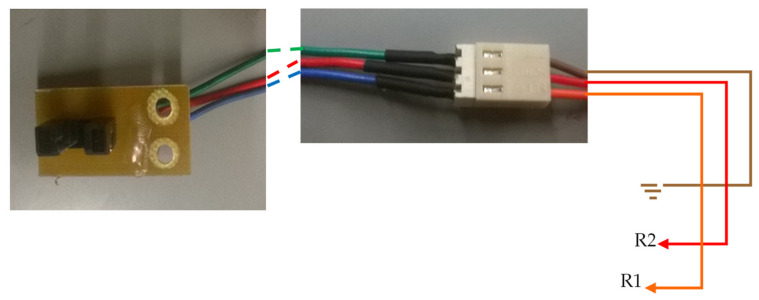
Image of the optical sensor and plug connection, connecting the board to the electrical circuit.

The voltage values used in the circuit shown above (VCC and GROUND) must correspond to the voltage values of the GPIO. In this case, since a Raspberry Pi 2B is used, the VCC value should be 3.3 V (the maximum voltage that the GPIO pins of Raspberry Pi support). Figure 15 shows an image with the optical sensor as well as the illustration of the connections between the optical sensor and the circuit of Figure 13.

**Figure 16:**
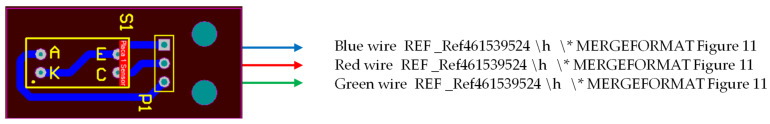
PCB design of an optical sensor board (bottom layer).

The PCB design for the optical sensor is shown in Figure 16. Due to the lack of space in some places where there is a need to place the optical sensors, it was decided to use a PCB (Figure 14) with several electrical circuits. Figure 16 illustrates how the connections of this circuit to the plugin Figure 15 are to be made.

#### 3.2.2. Mechanical Sensors (Waste Bin Drawer)

In addition to the optical sensors, switches were used as position sensors. When the switch is pressed, the position of the part in question is known. The switches have been chosen because of their low cost and the possibility of being used in places where ambient light can interfere with the optical sensors. As proof of the use of the switches as sensors, one was used to check the placement of the waste bin drawer. Table 2 shows the switch description used, as well as the reference to the annex with the datasheet.

**Figure 17:**
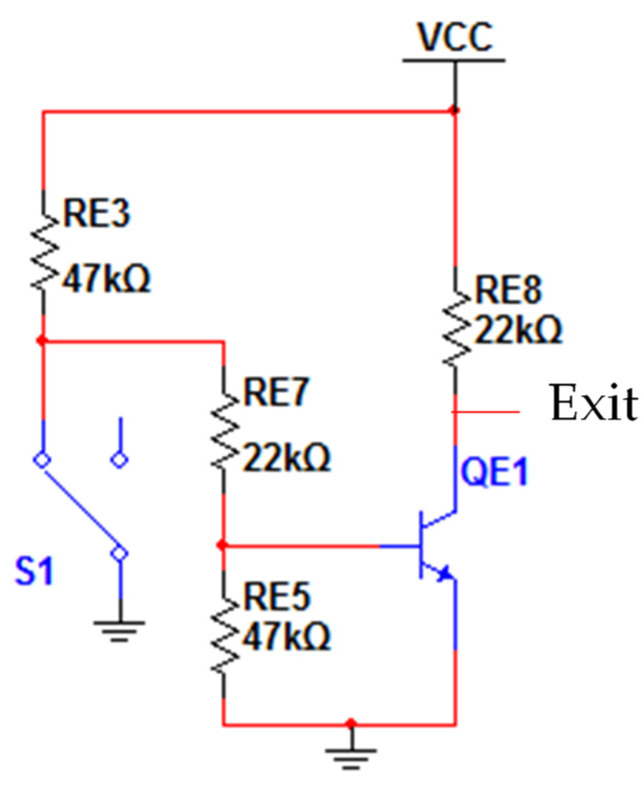
Circuit diagram of the waste bin sensor.

Figure 17 shows the wiring diagram used with the waste bin sensor.

The electrical circuit of Figure 17 is the same used for the optical sensors (Figure 13), except that the LED sensor branch of the optical sensor is left open. A switch with the contacts in the normally closed position was used as the drawer sensor. When the switch is not pressed, S1 (ground 0 V) is connected to resistor RE3 and RE7, causing the output of the electrical diagram to be the value of VCC (3.3 V) and indicating that the drawer is not inserted. If the switch is pressed (drawer inserted), the switch S1 changes state, making the ground pin (0 V) not be connected. This time, the output of the electric circuit has 0 V, indicating that the drawer is inserted in the equipment.

**Figure 18:**
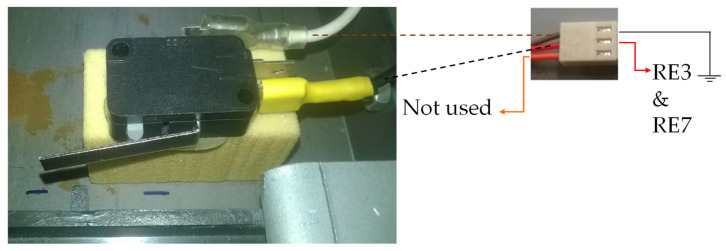
Waste bin sensor.

In Figure 18, an image of the switch used as the sensor of the drawer is shown, as well as an illustration of how the connections between the switch and the circuit of Figure 17 have been made.

#### 3.2.3. Electrical Power Scheme for Optical Sensors and Mechanical Sensors

**Figure 19:**
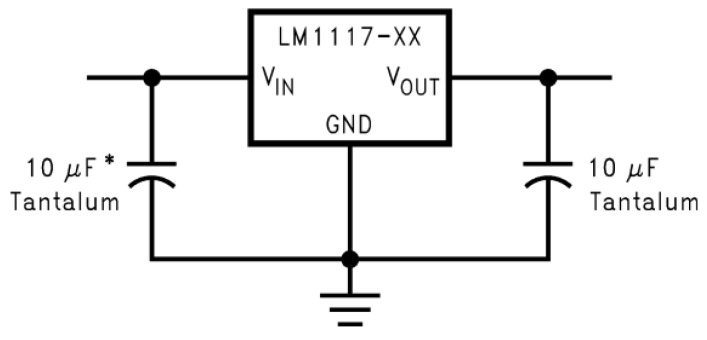
Conversion circuit for 5 V (V_IN_) to 3.3 V (V_OUT_).

As the control system (Raspberry Pi) only supports a voltage of 3.3 V on its GPIO pins, the various sensors of the BindTronic equipment were fed with this voltage value. The Raspberry Pi control system has a 3.3 V power supply pin, with a very low current limitation. In this way it was necessary to convert the 5 V present on the AC motor plate to the 3.3 V, using the voltage regulator LM1117T-3.3. Conversion from 5 V to 3.3 V could also be carried out through the 5 V pins present in Raspberry Pi, as these allowed for supplying the necessary current to the various sensors and circuits. In Figure 19, it shows the wiring diagram required to convert 5 V to 3.3 V using the voltage regulator LM1117T-3.3.

**Figure 20:**
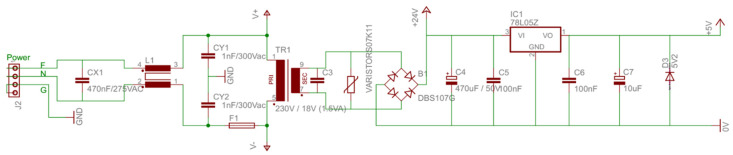
Electrical schematic of the AC motor power by the NC20 board, where 5 V is converted to 3.3 V by Figure 17.

Figure 20 shows the wiring diagram of the ACCO AC electric motor power board (used in other equipment), where the connection to the V_IN_ input of Figure 19 is made.

**Figure 21:**
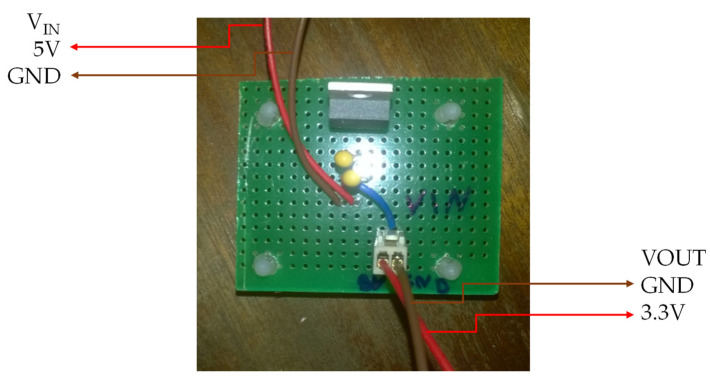
PCB board with an electric circuit for 5 V to 3.3 V conversion through the LM1117T-3.3 voltage regulator.

In Figure 21 it is possible to observe the PCB board from the Figure 19 diagram and the connector’s description. The VIN input connects the 5 V output in the diagram of Figure 20 and the VOUT output connects to the various electrical circuits that need to be supplied with 3.3 V (Figure 6 and Figure 15).

### 3.3. Engine Definitions and Schematics

In this section, we present the electric motors used in the BindTronic prototype, as well as the electrical diagrams used to control the motors. There are also images demonstrating the connections in the prototype.

#### 3.3.1. Spine Closing Motor

For the spine closing motor, a DC motor was used, and its description, reference and datasheet attachment are shown in Table 3. This motor was used because of its smaller dimensions when compared to AC motors of the same prices and characteristics.

**Figure 22:**
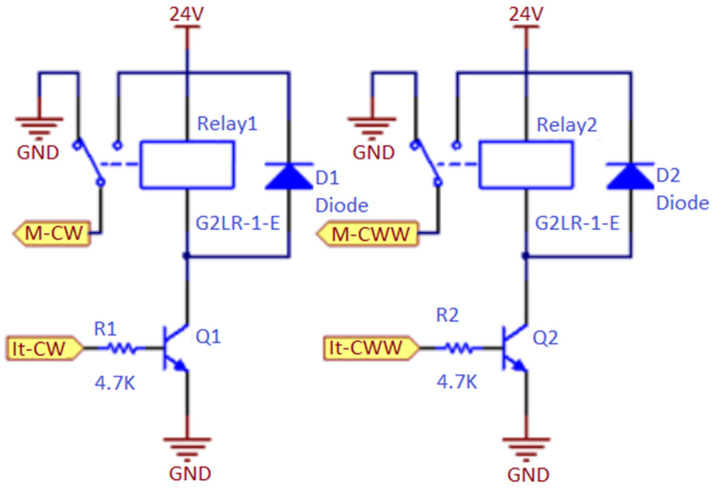
Electric scheme of the DC motor’s drive.

Figure 22 shows the electrical diagram used to control the DC motor.

This scheme was used by means of an electric motor, and two transistors that serve to activate the relays. By activating the transistors Q1 and Q2, it is possible to activate the relays and control the drive of the electric motor, such as the direction of rotation. If both transistors are active, the motor brakes; if no transistor is active, the motor is turned off.

**Figure 23:**
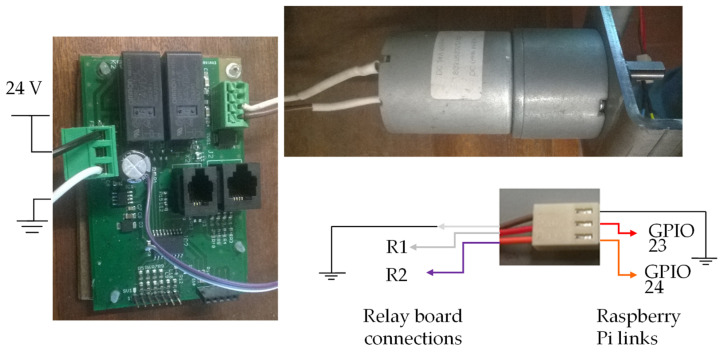
Image showing the connections between the spine closing motor and the control board, as well as the connections to the GPIO.

Figure 23 shows an illustration of the connections between the electric motor, the electric motor control pins, and the circuit board of Figure 22.

Figure 23 shows the connections made between the relay board and the Raspberry Pi through the three-pin connector on the board with the relays. In the cables of the electrical connector marked R1 and R2, a resistance of 4.7 KΩ was added between the plug and the board. If motors other than those in the prototype are used, it is necessary to consider the connections between the motor and the board with the relays so that the motor turns in the correct direction. See the description of the GPIO pins above in this document to help understand what the engine should do when the GPIO is activated (activating the engine).

#### 3.3.2. Document Height Adjustment Motor

For the document height adjustment motor, a DC motor was used, as described in Table 4. The document height adjustment motor has been chosen to have the same supply voltage as the spine closing motor.

The electrical diagram used to control the document height adjustment motor is the same for the control of the binding motor. This is possible since the motors used the same supply voltage (24 V).

**Figure 24:**
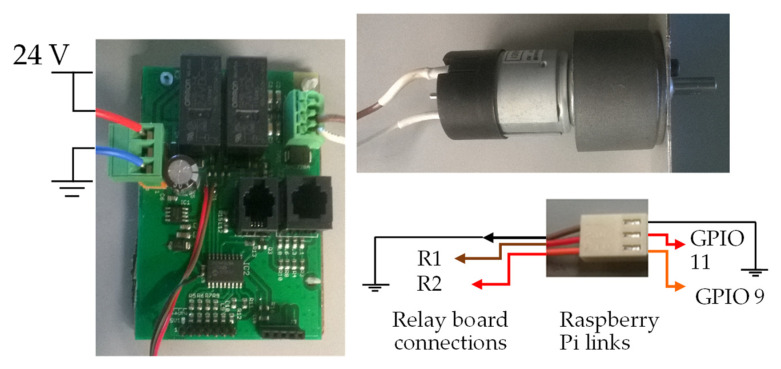
Image showing the connections between the document height adjustment and the control board, as well as the connections to the GPIO.

Figure 24 shows an illustration of the connections between the electric motor, the electric motor control pins and the circuit board of Figure 22.

Figure 24 shows the connections made between the relay board and the Raspberry Pi through the three-pin connector on the board. Connector cables marked R1 and R2 with a resistance of 4.7 kΩ were added between the plug and the board. If motors other than those in the prototype are used, it is necessary to consider the connections between the motor and the plate with the relays so that the motor turns in the correct direction.

#### 3.3.3. Document Punching Motor

For the document punching motor, an AC motor was used, with its description, reference, and annex presented in Table 5. This punching motor was chosen to have the necessary torque for punching the block of sheets.

**Figure 25:**
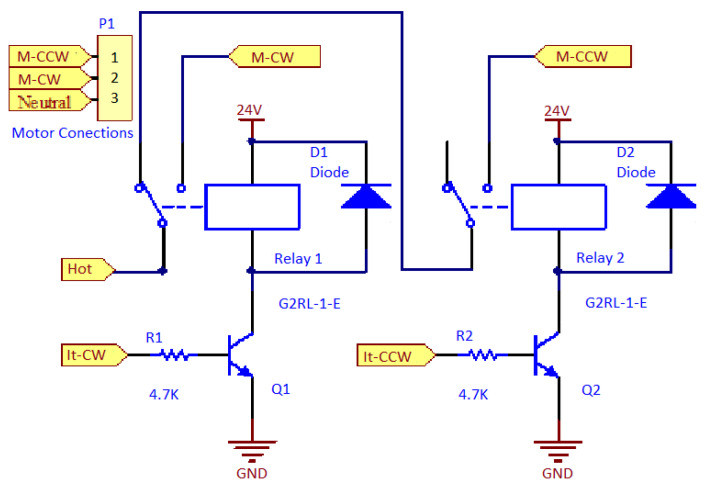
Electrical circuit diagram for the control of the punching motor document.

Figure 25 shows the electrical scheme used to control the punching motor of the document.

The electric circuit for the punching motor (Figure 25) is different from the electrical schematics for motors already shown, since the punching motor is AC. The pin marked as “neutral” is connected directly to the motor. The pin marked as “hot” passes through the circuit with the relays to set when the motor is driven, and the direction. This scheme is designed to prevent the hotwire from being connected simultaneously to the two connections of the electric motor.

**Figure 26:**
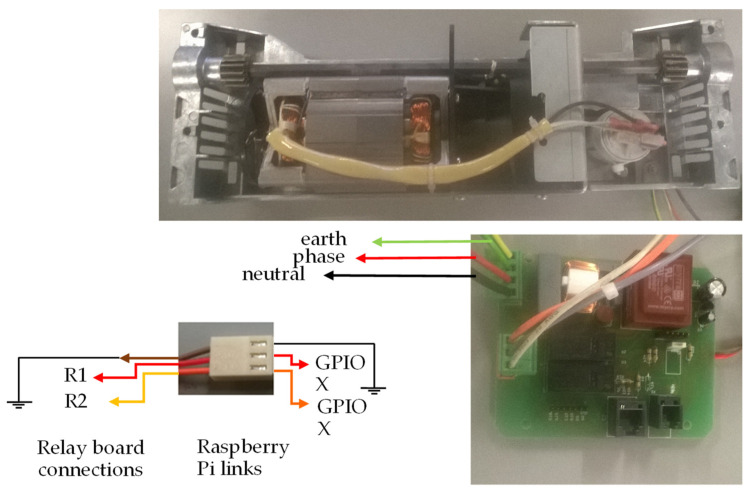
Image showing the connections between the punching motor and the control board, as well as connections to the GPIO.

Figure 26 shows an illustration of the connections between the electric motor, the electric motor control pins, and the circuit board of Figure 25.

The image in Figure 26 shows the connections made between the relay board and the Raspberry Pi through the three-pin connector on the board with the relays.

In the cables of the electrical connector marked as R1 and R2, a resistance of 4.7 KΩ was added between the plug and the board. If motors other than those in the prototype are used, it is necessary to consider the connections between the motor and the plate with the relays so that the motor turns in the correct direction. See the description of the GPIO pins above in this document to help you understand what the engine should do when the GPIO is activated (activating the engine). The plate of Figure 26 is different from that shown in Figure 23 and Figure 24 because this plate is used with an AC motor in contrast to the 24 V DC motors of Figure 23 and Figure 24.

### 3.4. Wiring Diagram of the Document Thickness Measuring Plate

**Figure 27:**
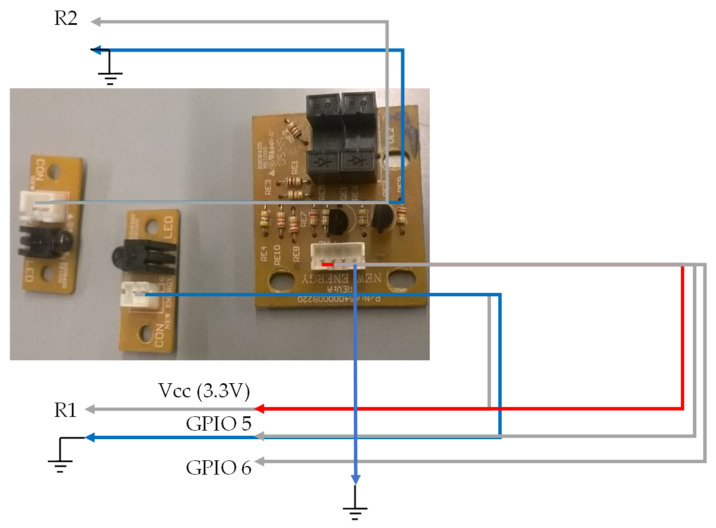
Document thickness measuring plate, with reference to the electrical connections.

For the measurement of the document thickness, the holder and sensors of the P300 binding equipment (Figure 27) were used.

Figure 27 shows the connections made to the electrical plates present in the document thickness measurement system. This system consists of three optical sensors. The first optical sensor’s LED and phototransistor photo are physically separated, as opposed to the other two optical sensors. To the right of the second image, we have the phototransistor that will connect the resistor R2 of the circuit (Figure 13). In the middle, there is the light emitter of the optical sensor (LED), which connects the resistance R1 of the same circuit of Figure 13. The circuit output of Figure 13, where the LED and the phototransistor of the first sensor of this system are connected, is connected to the GPIO 07 pin of the Raspberry Pi. The remaining two optical sensors already have their electrical circuits, similar to those in Figure 13.

The first sensor, which is composed of the LED and the phototransistor that are physically separated, is used to measure small-sized documents, as well as to verify if any document is inserted.

### 3.5. Wiring Diagram of the Inserted Spine Plate for Binding

**Figure 28:**
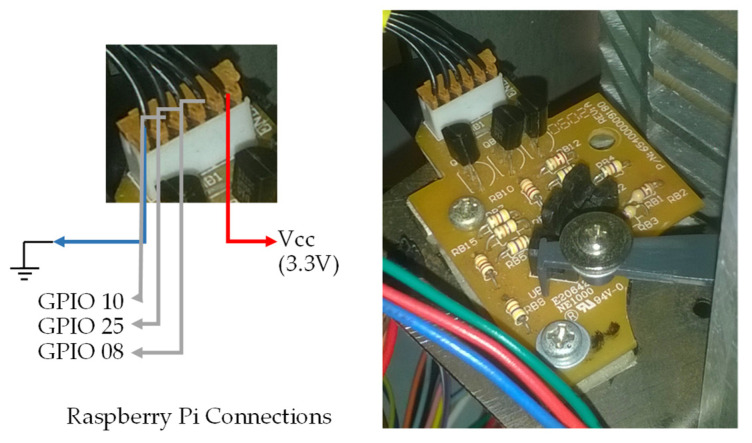
Measuring the spine board and the electrical reference connections.

For the measurement of the inserted spine for binding, the holder and sensor board of the binding equipment P3000 (Figure 28) were used. This plate was placed so it does not interfere with the binding, but allows the measurement of the spine inserted in the prototype.

The electrical board of Figure 28 has three optical sensors, and the electrical circuit being used is the same as shown in Figure 13. In this board, the electrical circuit is already present, and it is not necessary to use the circuits present in the board of Figure 14. Figure 28 also illustrates the connections made to this board.

### 3.6. Document Thickness Measurement System with the Operating Principle of a Potentiometer

**Figure 29:**
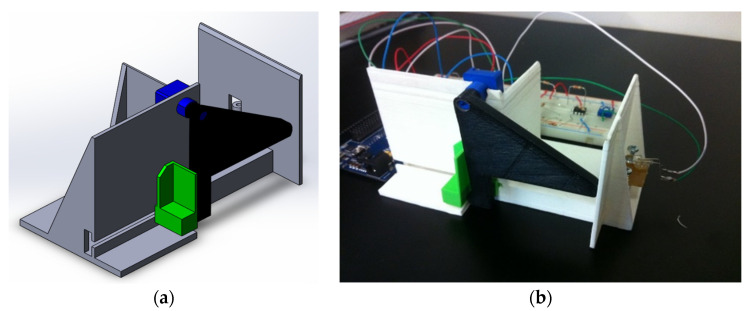
System for measuring the thickness of a document by the principle of a potentiometer: (**a**) the CAD model; (**b**) the prototype developed by 3D printing.

For the measurement of all document thicknesses corresponding to the various sizes of ProClick, wire and comb spines, a system based on a potentiometer was also developed (Figure 29).

The system of Figure 29 uses a cam coupled to the potentiometer to change its resistance according to the rotation of the cam. The cam undergoes rotational movement when a document is placed at the measurement site. To ensure that the document is flush with the correct location and that the cam returns to the starting position, a spring is applied that applies force to the green part of Figure 29. The mechanical working principle of this system is similar to that used in the BindTronic prototype and is only used as an optical sensor to check when a document is inserted, as well as to measure the smallest thickness of the document.

**Figure 30:**
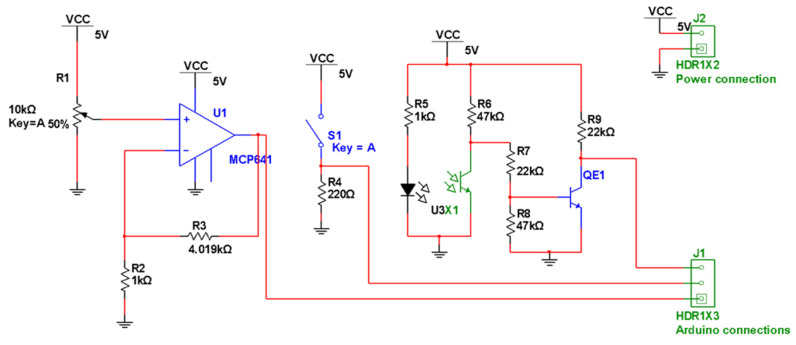
Electrical diagram of the circuit for measuring the thickness of a document with the operating principle of a potentiometer.

Figure 30 shows the electrical scheme developed for this system.

Circuit 1 of Figure 30 shows the electrical scheme where the potentiometer is connected, which measures the thickness of the documents. Due to the small variations of the desired voltages for the measurement of document thickness, a non-inverting amplifier circuit was used to amplify the signal to be measured. The non-inverting amplifier circuit was calculated to match the measurement range between the ADC (analog to digital converter) measurement values. The ADC used can measure between 0 and 5 V and differentiate between 1024 different values, having a sensitivity that is around 5 mV. The ADC used is that of Arduino, and it is necessary to connect circuit 1 to an Arduino GPIO pin that has an ADC.

Circuit 2 of Figure 30 was developed as a simple push-button to indicate to the program when the values were to be measured.

Circuit 3 of Figure 30 is a circuit similar to that of Figure 13, and this system also uses an optical sensor for measuring the smallest document thickness corresponding to the lowest capacity of a spine, as well as checking for any document inserted in the measuring system.

Two versions of the software are available with this document. One of the versions is prepared to run on the Windows operating system, whereas the other version is prepared to run on the Raspbian operating system found in Raspberry Pi. The difference between the two versions is minimal, and the Windows version has only commented on the creation of the variables/objects that access the GPIO pins, their definition as well as all access to these variables. To be able to simulate the program in Windows, these variables, instead of being a “GPIOPinDriver” object to “interact” with the GPIO, are variables of type “int” (integer) that receive the value “0” or “1” (for example, compare the code in the “BindWindow” class of the two versions).

The program was developed in the C# programming language and the GTK libraries are used for the graphical component. In this way, it is possible to develop and run the prototype software on the three major operating system platforms that currently exist: Windows, Mac OS X and Linux.

## 4. BindTronic Control Software

### 4.1. Working Block Diagram of the Prototype

**Figure 31:**
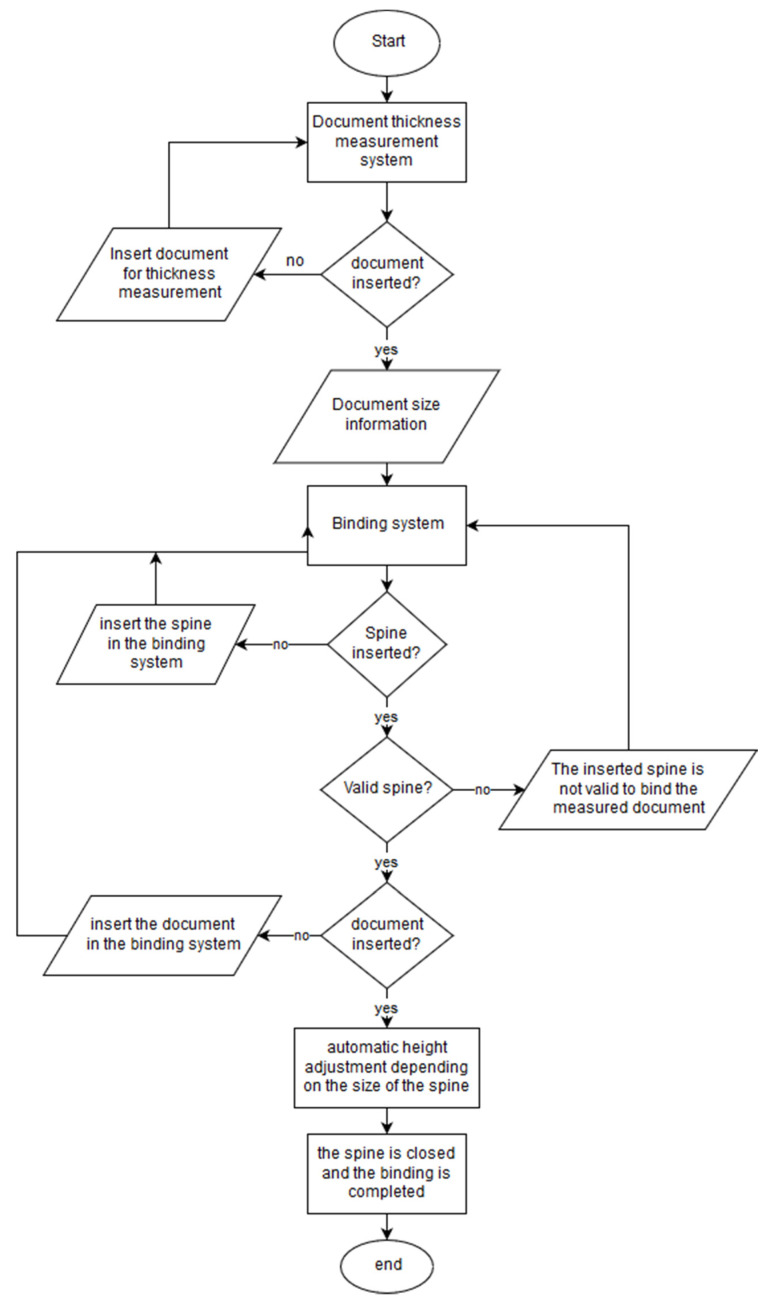
Block diagram for the binding of a document.

The block diagram for binding is shown in Figure 31. The block diagram of the binding and punching are presented separately for a better understanding. The operation of both is isolated, and through the flowchart, it is possible to verify how to choose one action or another, and how the program developed for each action.

The binding block diagram begins with the document thickness measurement. The system waits for a document to be entered for measurement, informing the user of the document size that corresponds to an existing spine size. Although the document is not entered for measurement, the program prompts the user to insert a document and waits. After measuring the document and information on its size, the program passes to the binding system. Here, it is checked whether any spine is inserted, using the mechanism of measuring the inserted spine, and advising the user to insert a spine. When a spine is inserted, the same mechanism measures the size of the spine inserted. In this way the control system can validate whether the inserted spine allows the binding of the measured document or not, and informs the user. After spine validation, the program checks if there is any document inserted for binding (only the developed graphical component), thus avoiding binder errors (spine closure without documents) and warning the user if the document is not present in the correct place. After all validations have passed, the document height adjustment mechanism is activated, raising the document to the correct height to be bound with the inserted spine. After raising the document, the spine closing mechanism is engaged, closing the spine while inserting the spine fingers into the holes in the document.

With the software developed, it is not obligatory to measure the size (thickness) of the document, and the user can skip this step. If you do, the program asks you which bump size to use for binding, and you need to choose a size from the existing ones. In this case, the remaining operation of the block diagram is the same, and the validation of the spine is performed only by checking whether the inserted spine is equal to that which was chosen. In this mode, it is not possible to bind documents with spines higher than the chosen one, but only with spines equal to the one chosen. At any point in the program, up to the start of the motors for binding (document height adjustment and buckle closing), you can go back and modify the actions and options you have taken.

**Figure 32:**
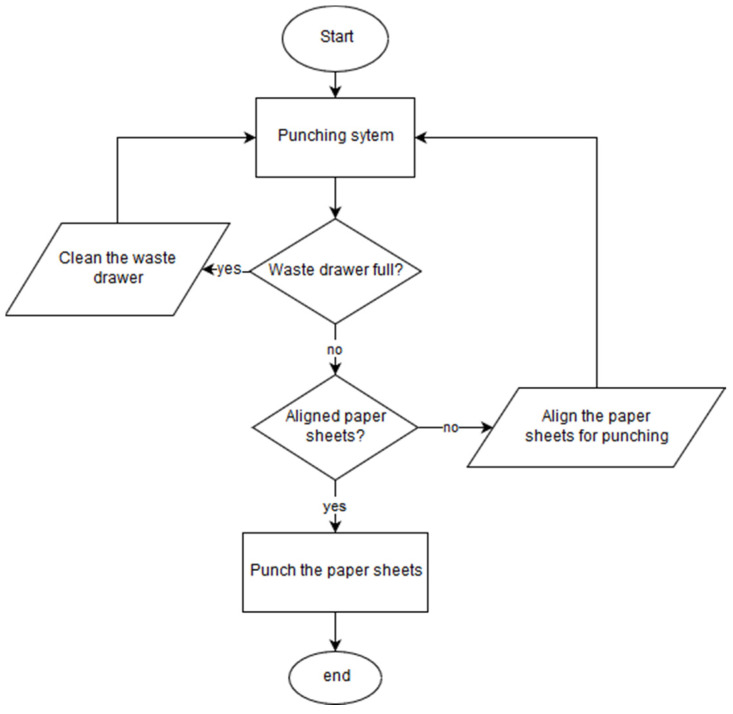
Block diagram for punching a block of sheets.

The block diagram for punching is shown in Figure 32, and the punching diagram operation is simpler than the binding diagram and does not require any information from the systems and mechanisms used for the binding. Thus, it is possible to say that the punching system is only connected to the rest of the prototype by the control system and its interface, allowing the user to choose the actions he/she wants to perform (punching, binding, etc.).

The punching diagram begins when the user chooses the program that wants to perform punching. It is then checked if the waste bin is inserted and full, telling the user if it is necessary to empty the waste bin. Checking if the drawer is full is accomplished by counting the number of perforations since the drawer was last cleaned. If the user chooses not to use the waste tray in the options, a check is performed to verify that the drawer is inserted, prompting the user to remove it.

In addition to checking the waste tray, the alignment of the block of sheets to be perforated (only the graphical component has been developed) is also verified. If the block of sheets is not inserted or aligned, the user receives information to insert or align the document. Similar to what happens with the waste drawer; you can choose from the software options that you do not want to use sheet alignment for punching. In case the alignment is not used, no type of verification is performed in relation to the block of sheets that is to be perforated. After the software passes through the two checks in the punching diagram (Figure 32) the program allows punching to be performed, and the driller decides when to carry it through the touchscreen.

### 4.2. Software Features Implemented in the Prototype

Several software features have been developed to make the prototype easy and comfortable to use, as have the features that are necessary to compete with today’s binding equipment. The main relevant characteristics that constitute the BindTronic prototype are as follows:

- Several languages available;
- Easy addition of more languages;
- Counting of perforations and bindings (totals and chains);
- Reset of current counts;
- Possibility of using or not using the waste tray;
- Reset the capacity of the waste tray automatically when you remove it (5 s);
- Possibility of using or not using the alignment of the sheets (graphical component only);
- Using a screensaver;
- Possibility of changing the time for the screensaver’s performance;
- Automatic document measurement;
- Automatic and autonomous measurement of the spine inserted in the binding equipment;
- Automatic closing of spines;
- Automatic and autonomous adjustment of the height of the document to be bound according to the inserted spine;
- Verification of whether the spine allows binding, warning the user if it does not;
- Return to the initial position of the binding system in the case of failure and the loss of its location (at the beginning of the program, or when binding, if there is a spine inserted and to be measured);
- Possibility of using the equipment without measuring the document (choosing the size of the spine to be used);
- Automatic sheet perforation;
- Return the initial position of the punching system in case of failure and the loss of its location (it is necessary to re-bind, but the system only returns the initial position this time);
- Stopping the punching system, if the blade descends more than 1 s or the blade rises for more than 1 s (fault protection); and
- Class associations.

**Figure 33:**
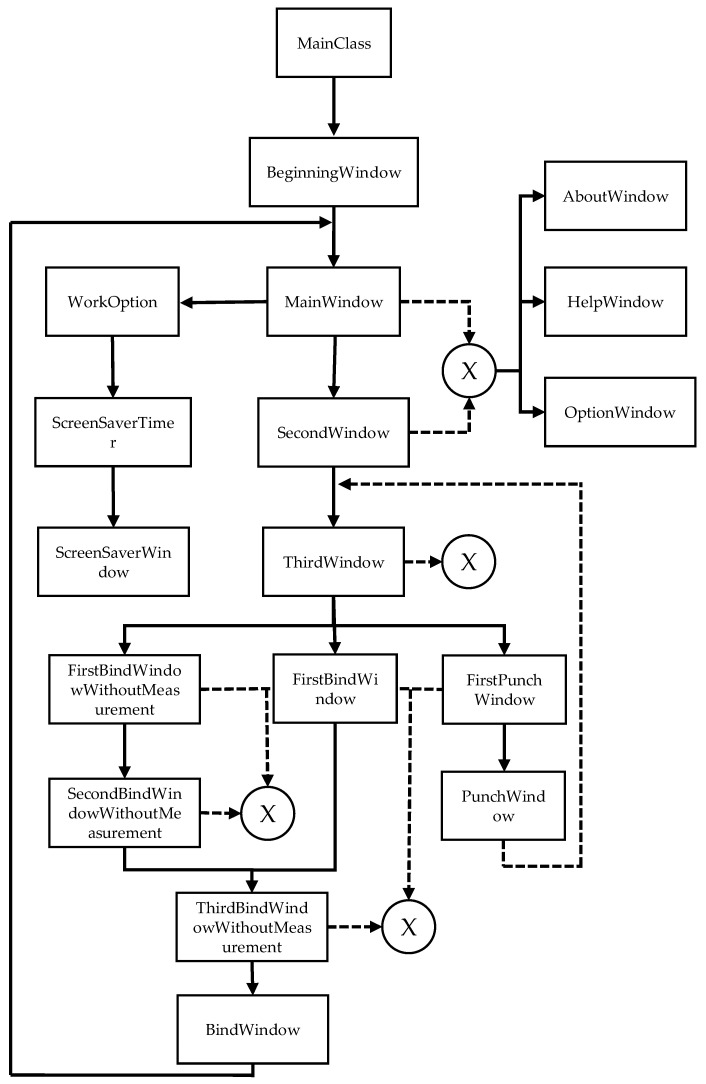
Association between program classes.

In Figure 33, it is possible to observe the association between the classes of objects. To this association, we can also call the order of creation/use of the classes during the operation of the program. When there is an association between classes, it means that the previous class has an object of the next class, creating it in this way. In the case of the BindWindow and PunchWindow classes, they do not have any objects of another class, but when they are destroyed, the program destroys the previous classes until the MainWindow and ThirdWindow classes, respectively.

The classes that are binding to X are linked by X through inheritance. That is, these classes can create objects of the AboutWindow, HelpWindow, and OptionWindow classes because they receive the objects and methods through the inheritance of the MatherWindow class (not shown in Figure 33). In the documentation provided, in addition to the diagram in Figure 33, a UML diagram and a flowchart of the prototype operation are also provided.

## 5. Conclusions

The designed and created prototype is nearly fully automatic, requiring the user to only feed paper sheets on the three advanced systems and choose the actions for execution on a touchscreen. The activities of the user are always monitored and verified to guarantee a perfectly punched and bound document. In terms of usability, major improvements are anticipated, which is a major problem when using this sort of equipment. Because of the minor adjustments required to bind a document and inaccurate spine selection, users tend to create errors that can be avoided with the implemented algorithm, sensors and user interface. Due to the automatic movements and instruction supplied by the touchscreen, the binding cycle is expected to significantly decrease. The presented developments are expected to substitute the current automatic punch and bind equipment of the company’s portfolio. This prototype will be researched and enhanced in future work taking into consideration the ergonomic requirements for its best use. Furthermore, finite element software simulation will be carried out to improve the geometry and punching mechanism of the blade, with the aim of improving the punching capacity without compromising reliability.

## 6. Patents

The work reported in this manuscript resulted in a national patent nr. 108647.

## Figures and Tables

**Table 1:** Optical sensor characteristics.

Manufacturer	Reference	Type
OMRON	EE-SX1081	Transmission

**Table 2:** Characteristics of the waste bin mechanical sensor.

Reference	Type	Contact Format
D3V	Reliable Basic Switch with External Lever	SPDT

**Table 3:** Characteristics of the spine closing motor.

Reference	Power Voltage [V]	rpm
SG37RS35ZY40	DC—24 V	50

**Table 4:** Characteristics of the document height adjustment motor.

Manufacture	Reference	Supply Voltage [V]	Rated Speed [rpm]
Mclennan	1308-24-250	DC—24	8

**Table 5:** Characteristics of the punching motor.

Reference	Power Supply [V]	Rpm for 60 Hz	Rpm for 50 Hz
ACC8055Ea-70JB150G10	230 V AC	23 ± 2	20 ± 2

## References

[1] Figueiredo L., Sousa J., Machado J., Mendonça J.P. Optimized punches geometry for paper punching systems: An industrial approach. Proceedings of the 2017 4th International Conference on Control, Decision and Information Technologies (CoDIT).

[2] Sousa J., Figueiredo L., Machado J., Mendonca J.P., Hamrol A., Ciszak O., Legutko S., Jurczyk M. (2018). Measuring the Punching Profile of a Punch And Bind Machine. Proceedings of the Advances in Manufacturing.

[3] Sousa J., Pinho T., Figueiredo L., Mendonça J., Machado J. (2015). Development and Optimization of a Paper Punching System. Proceedings of the International Mechanical Engineering Congress and Exposition.

[4] Figueiredo L., Sousa J., Monteiro L., Mendonça J., Machado J. (2016). Innovative Mechatronic Approach to Redesign a Punch and Bind Machine. Advanced Manufacturing.

[5] Cox S. (1997). Binding Machines. Patent.

[6] Badham G., Morgan G.O. (2007). Binding Machine for Closing Wire Comb Binding Elements. Patent.

[7] GBC ClickBind Binding Spines|GBC. https://www.gbceurope.com/en-ax/products/gbc-clickbind-binding-spines_387302e.

[8] Sakata T., Yoshie T. (2005). Binder and Binding Device. Patent.

[9] Knight C. (2004). Binding Machine and Method. Patent.

[10] Jervis R., James M., Hadden R. (2009). A Document Binding Machine. Patent.

[11] Finishers│GBC—GBC Connect. https://www.gbcconnect.com/us/us/2448/finishers.

[12] Vogel-Heuser B., Hess D. (2016). Guest Editorial Industry 4.0–Prerequisites and Visions. IEEE Trans. Autom. Sci. Eng..

